# Comparison of swab types for collection and analysis of microorganisms

**DOI:** 10.1002/mbo3.1244

**Published:** 2021-11-15

**Authors:** Natalie M. Wise, Sarah J. Wagner, Travis J. Worst, Jon E. Sprague, Crystal M. Oechsle

**Affiliations:** ^1^ Ohio Attorney General’s Center for The Future of Forensic Science Bowling Green State University Bowling Green Ohio USA

**Keywords:** DNA recovery, microbial DNA yield, microbial forensics, microbiome, qPCR, sampling substrate, swab composition

## Abstract

The human microbiome has begun to emerge as a potential forensic tool, with varied applications ranging from unique identification to investigative leads that link individuals and/or locations. The relative abundance of the combined DNA of the microbiome, compared to human nuclear DNA, may expand potential sources of biological evidence, especially in cases with transfer or low‐copy number DNA samples. This work sought to determine the optimal swab type for the collection and analysis of microorganisms. A bacterium (*Proteus mirabilis*) was deposited by pipette onto four swab types (cotton, flocked, dental applicators, and dissolvable), and extraction and real‐time PCR quantitation of the bacterial DNA were performed, which allowed for absolute microbial DNA recovery and comparison of yields across the four sampling substrates. Flocked swabs had the highest yield (~1240 ng) compared to the cotton swabs (~184 ng), dental applicators (~533 ng), and dissolvable swabs (~430 ng). The collection efficiency was further evaluated for cotton and flocked swabs using dried microbial samples spotted onto non‐porous surfaces (treated wood, glass, plastic, and tile). Flocked swabs performed consistently better across wood, glass, and tile, but showed decreased recovery from plastic. The cotton swabs failed in the recovery of *P*.* mirabilis* DNA across all surfaces. Knowing the appropriate sampling substrate will be useful as others continue to investigate the use of the microbiome as a forensics tool.

## Graphical abstract

The human microbiome is an emerging tool for forensic investigations, but its use could be hindered by improper collection at crime scenes. Here, we show that flocked swabs outperform cotton, dissolvable, and dental applicators in their ability to collect and release *Proteus mirabilis* DNA for analysis. Therefore, the ubiquitous use of cotton swabs for biological sample collection should be re‐evaluated.

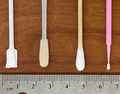

## 1 INTRODUCTION

In recent years, the human microbiome, or the collective term for all the microorganisms living on or within the human body (NIH Human Microbiome Project), has begun to emerge as a potential forensic tool. Forensically, the microbiome has the potential to be utilized as a unique identifier (Fierer et al., 2010; Oh et al., 2016), to link cohabiting individuals (Song et al., 2013), or to connect a person with a location and/or an object to a person (Yatsunenko et al., 2012). The successful use of the microbiome, regardless of the purpose, will involve the collection of the biological material from a surface, followed by its subsequent release from the collection substrate, and analysis. Much research has been conducted on the optimal sampling substrate for use in traditional forensic DNA analysis and body fluid identification (Adamowicz et al., 2014; Luna, 2017; Viviano et al., 2018; Voorhees et al., 2006); however, the matter has not yet been extensively studied for the collection of the microbiome. Swabbing and tape‐stripping are comparable methods for sampling the microbiome (Ogai et al., 2018), but the potential inhibitory effects that adhesives can have on DNA extraction and downstream PCR amplification may make swabbing preferable. However, different types of swabs hold and release biological material differently (Bruijns et al., 2018). The objective of this study was to determine the optimal swab type for collection and analysis of the microbiome by comparing traditional cotton, nylon flocked, dissolvable swabs, and dental applicators. Despite being inefficient at releasing biological material during extraction processes (Adamowicz et al., 2014; Bruijns et al., 2018; Viviano et al., 2018; Voorhees et al., 2006), cotton swabs are widely available and used by the forensic community, even though other swab types may lead to better sample recovery. With perpendicular fibres and no internal mattress core, flocked swabs are designed for the effective collection and elution of samples (COPAN Diagnostics Inc, 2020). Dissolvable swabs, made from cellulose acetate, are soluble in buffers that contain chaotropic salts, like guanidinium thiocyanate used in commercially available nucleic acid extraction kits (Luna, 2017). Dental applicators can be brushes of various sizes but are typically composed of non‐absorbent nylon flocking adhered to a spherical tip and used in the dental and make‐up industries to apply various products (Safeco Dental Supply, 2020). Given the differences in the microbiome, and associated microbial DNA, compared to human nuclear DNA, it is also unknown if surface or sub‐surface interactions will be similar (Alketbi & Goodwin, 2019; Verdon et al., 2013; Wood et al., 2017). Thus, the collection efficiency from non‐porous surfaces using the flocked and cotton swabs was also evaluated.

## 2 MATERIALS AND METHODS

### 2.1 Swab preparation

**FIGURE 1:**
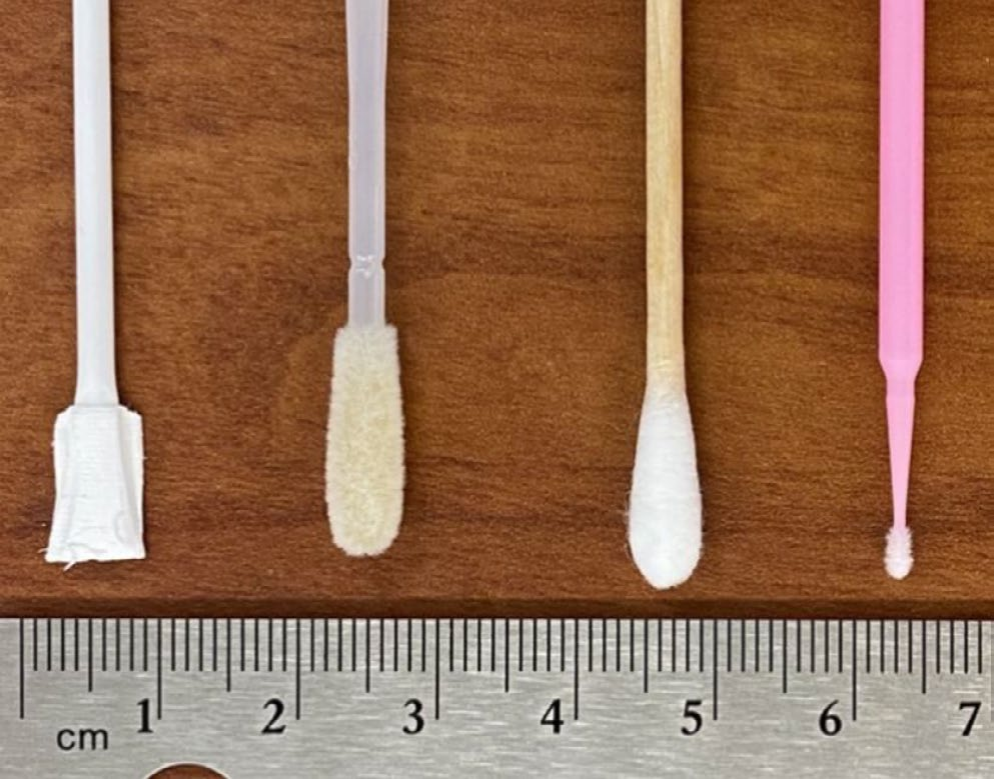
Swab types assessed for microbial DNA recovery. From left to right: Luna Innovations Incorporated™ dissolvable swabs (unshaven), Copan FLOQSwabs™, Puritan™ 6″ Standard Cotton Swab w/Wooden Handle, and Plasdent™ Maxapplicator™ ‘Regular Size’ (2.0 mm) Dental Applicators.

*Proteus mirabilis* is a bacterial species typically found in the gut microbiome and was used here as an available and representative bacterial component of the human microbiome. *Proteus mirabilis* was cultured, collected, washed, and pelleted via centrifugation to create a stock. Through prior quantitation and experimentation, it was determined that 10 µL of the uniformly mixed stock should result in DNA yields within the dynamic range of the qPCR standard curve (Wagner, 2021). The *P*. *mirabilis* stock was deposited onto eight replicates of each of four swab types: Puritan™ 6″ Standard Cotton Swab w/Wooden Handle (Puritan™), Copan FLOQSwabs™ (Copan), Plasdent™ Maxapplicator™ ‘Regular Size’ (2.0 mm) Dental Applicators (Safco Dental Supply LLC), and dissolvable swabs (Luna Innovations Incorporated™; Figure 1). Dental applicators were included due to their relatively small volume because it was thought that there may be fewer places for biological material to become trapped within the swab material (Safeco Dental Supply, 2020). In this case, the dental applicators used had a 2.0 mm head, while the traditional cotton swabs used were ~10.0 mm in length. The brand of dissolvable swabs used here was in development. According to the manufacturer, the provided prototypes contained ~20 mg total cellulase acetate fibre (pictured unshaven in Figure 1), but the fibre material entering the extraction reaction should be minimized to 5–7 mg, which required manual shaving of the dissolvable swabs to ~¼ the original size before use. As a positive control, eight replicates of the same *P*. *mirabilis* stock were added to sterile microcentrifuge tubes without a swab substrate. A negative control, consisting of a sterile microcentrifuge tube serving as a reagent blank, was included in each round of extraction and processed in the same manner as test samples.

### 2.2 Surface preparation and swabbing

Non‐porous surfaces utilized for sample collection included: treated wood flooring (Bruce American Originals Natural Oak Solid Hardwood Flooring, AHF Products), glass (8 in. ×10 in. ×0.125 in., The Home Depot, Inc.), plastic polypropylene plate (10.5″, Room Essentials™, Target Corporation), and tile (Grade 1, 3 in. ×12 in. × ¼ in., TrafficMaster Laguna Bay Glazed Ceramic Bullnose Floor and Wall Tile, Shaw Industries, Inc.). Surfaces were cleaned twice with a 10% bleach solution and twice with Peroxide Multi‐Surface Cleaner and Disinfectant (8% H_2_O_2_; EcoLab). Each surface was sectioned into 3 × 3 cm^2^; 10 µL of the same *P*. *mirabilis* stock described above was spotted onto each section and allowed to dry (~2 h), with seven 3 × 3 cm^2^ on each surface designated for swabbing with flocked swabs and seven designated for swabbing with cotton swabs. The appropriate single swab was pre‐moistened with sterile, deionized water (~100 µl) and used to collect the *P*. *mirabilis* from the corresponding surface section. Swabbing was performed by applying firm and constant pressure and repeatedly rotating the swab throughout, for 30 s. After drying (~2 h), swab heads were removed from the sticks and transferred to 2.0 ml microcentrifuge tubes for storage at −10°C until further processing. A positive manipulation control, consisting of *P*. *mirabilis* spotted onto cotton or flocked swabs, and negative control, consisting of a sterile microcentrifuge tube serving as a reagent blank, were included in each round of extraction and processed in the same manner as test samples.

### 2.3 DNA extraction

Bacterial DNA was extracted using the Applied Biosystems™ MagMAX™ DNA Multi‐Sample Ultra 2.0 Kit (Thermo Fisher Scientific) and the manufacturer's suggested protocol (MagMAXTM DNA Multi‐Sample Ultra 2.0 Kit). This procedure was modified in that volumes were doubled for the dissolvable swab samples and negative controls following a recommendation provided by Luna Innovations Incorporated™. The purified DNA was eluted in 50 µL of MagMAX™ Elution Buffer.

### 2.4 DNA quantitation

All sample and control extracts (2 μl per sample) were quantified using 12.5 μl of iTaq™ Universal SYBR® Green Supermix (Bio‐Rad Laboratories Inc.), 1 μL of 10 µM 16S rRNA Forward ReadyMade™ Primers (Integrated DNA Technologies, Inc.), 1 μl of 10 µM 16S rRNA Reverse ReadyMade™ Primers (Integrated DNA Technologies, Inc.), and 8.5 μl of Invitrogen™ UltraPure™ Distilled Water (Thermo Fisher Scientific) in a Life Technologies MicroAmp® Optical 96‐well 0.2 ml Reaction Plate (Applied Biosystems). The forward primer sequence for the 16S rRNA Forward ReadyMade™ Primers was: AGA GTT TGA TCC TGG CTC AG, and the reverse primer sequence for the 16S rRNA Forward ReadyMade™ Primers was: ACG GCT ACC TTG TTA CGA CTT. *P*. *mirabilis* stock was extracted using the method described above, pooled, and quantified via NanoDrop (ThermoFisher Scientific) for use in a five‐point calibration curve ranging from 50 to 0.005 ng/µl. The Applied Biosystems™ QuantStudio™ 5 Real‐Time PCR System (Thermo Fisher Scientific) with the associated Design and Analysis Software v1.5.1 was used for quantitation. The thermal cycling parameters for the reaction were Ramp Speed 1.6°C/s, Hot Start 95°C for 4 min, and 35 PCR Cycles of 95°C for 10 s, 57°C for 30 s, and 68°C for 30 s. The Melt Curve Scheme was 95°C for 10 s, followed by a 65°C–95°C gradient (0.15°C/s).

### 2.5 Data analysis

Using the qPCR determined concentrations, total mass (in nanograms) of DNA recovered from each extract, averages, standard deviations (*SD*), and standard error of the means (SEM) were calculated across each swab type using Excel® (Microsoft, version 2101). Statistical analysis was performed using the statistical software R (v 4.0.2). Between‐group comparisons were conducted by ANOVA (*α* = 0.05) followed by a Tukey's Honest Significant Difference (HSD) test, as warranted.

## 3 RESULTS

**FIGURE 2:**
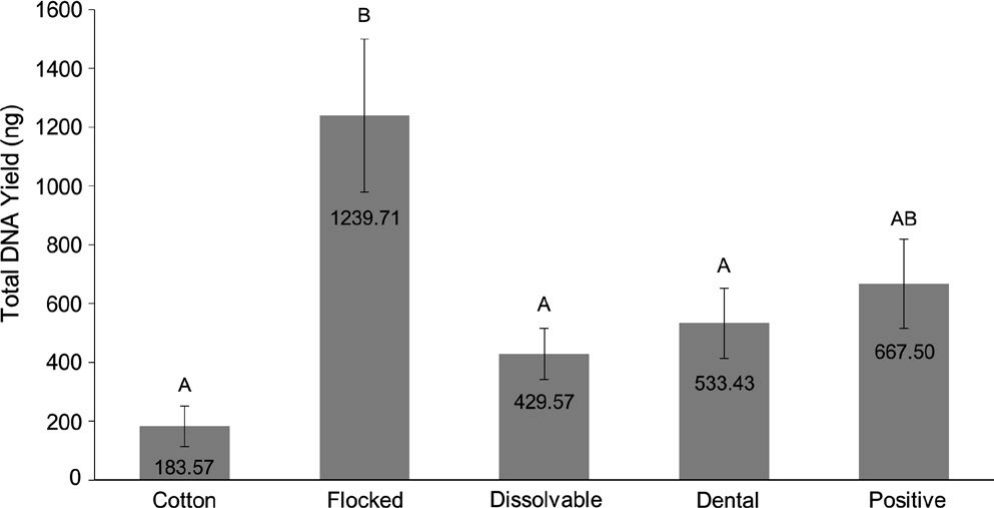
Total bacterial DNA yield following the direct deposit of sample onto the swab for each swab type tested (cotton, flocked, dissolvable, and dental applicators) compared to the positive manipulation control. Results displayed represent the average of the eight trials, with outliers removed, ±SEM. Letter designations represent Tukey's HSD comparisons: the same letter designation means results are not statistically different; when letter designations differ between groups, the *p*‐value is < 0.05.

There was a statistically significant difference in the total mass of microbial DNA recovered between the four swab types (*p *< 0.001). Flocked swabs had the highest yield (~1240 ng) compared to the cotton swabs (~184 ng, *p *< 0.001), dental applicators (~533 ng, *p *= 0.020), and dissolvable swabs (~430 ng, *p *= 0.006) (Table 1 and Figure 2). No statistical difference was observed between any of the swab types and the positive manipulation control. Using the whole microbe for sample preparation, rather than pre‐extracted genomic DNA, meant that starting microbial DNA concentrations were unknown, and a true before and after comparison of DNA concentration presented as the percent recovery for each swab type was not possible. As the positive control was not internal, normalizing DNA concentrations to the positive control values is not appropriate. However, because all samples came from the same well‐mixed *P*. *mirabilis* stock, the assumption can be made that equal starting volumes should have had similar starting amounts of microbial material. Making that assumption, it is appropriate to compare the average microbial DNA mass recovered from each swab type and the positive control, and those values are listed as percentages in Table 1.

**TABLE 1:** Total bacterial DNA yield from each swab type tested (cotton, flocked, dissolvable, and dental applicators) compared to the positive manipulation control.

	Cotton (ng)	Dental (ng)	Flocked (ng)	Dissolvable (ng)	Positive (ng)
	(8066)	(1948)	(3950)	(6849)	(9423)
	30	159	249	147	195
	544	473	1125	541	969
	100	373	2183	300	975
	246	1106	968	632	(3527)
	16	698	2047	135	198
	233	636	780	589	854
	116	289	1326	663	814
Average Mass (ng)	183.57	533.43	1239.71	429.57	667.50
Std. Dev.	182.33	314.85	686.63	229.75	370.24
SEM	68.91	119.00	259.52	86.84	151.15
Recovery Compared to Positive Control	27.50%	79.91%	185.72%	64.35%	–

Note

Values in parentheses were removed from the analysis as outliers. Results displayed represent the average of the eight trials, with outliers removed; one Std. Dev.; and SEM.

**FIGURE 3:**
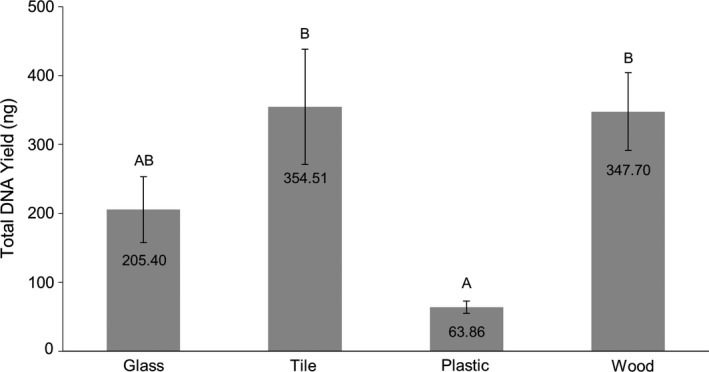
Total bacterial DNA yield from flocked swabs used to collect samples across four surface types (glass, tile, plastic, and wood). Results displayed represent the average of the seven trials, with outliers removed, ±SEM. Letter designations represent Tukey's HSD comparisons: the same letter designation means results are not statistically different; when letter designations differ between groups, the *p*‐value is <0.05.

For the surfaces sampled with flocked swabs, tile had the highest microbial DNA yield (~355 ng) compared to treated wood (~348 ng, *p *= 0.999), glass (~205 ng, *p *= 0.307), and plastic (~64 ng, *p *= 0.011) (Table 2 and Figure 3). Across the surface types, there was a substantial decrease (−72% treated wood, −83% glass, −95% plastic, and −71% tile; Table 2) in the amount of microbial DNA recovered between the bacterial samples spotted directly onto flocked swabs and those swabbed from surfaces using flocked swabs. Notably, all glass, tile, plastic, and six of seven wood samples swabbed with the cotton swabs yielded negative results, that is, no detectable bacterial DNA was recovered. On the one cotton swab of wood where amplifiable bacterial DNA was detected, the total quantity was 26.05 ng. Similarly, all reagent blanks except one yielded negative results, with the one reagent blank having a total quantity of 15.00 ng of DNA detected.

**TABLE 2:** Total bacterial DNA yield from flocked swabs used to collect samples across four surface types (glass, tile, plastic, and wood) compared to bacterial deposition directly onto flocked swabs.

	Glass (ng)	Tile (ng)	Plastic (ng)	Wood (ng)
	63	470	102	407
	360	212	(8)	505
	295	112	76	506
	(841)	784	50	348
	157	290	63	371
	256	366	42	155
	101	246	51	143
Average Mass (ng)	205.40	354.51	63.86	347.70
Std. Dev.	116.53	220.99	22.06	148.95
SEM	47.58	83.53	9.01	56.30
Recovery Compared to Direct Deposition on Flocked Swabs (Table 1)	−83.43%	−71.40%	−94.85%	−71.95%

Note

Values in parentheses were removed from the analysis as outliers. Results displayed represent the average of the seven trials, with outliers removed; one Std. Dev.; and SEM.

## 4 DISCUSSION

While not currently as individualizing as other traditional forensic methods, the microbiome has begun to emerge as a potential forensic tool. Due to the relative abundance of bacteria and other microorganisms compared to human nuclear DNA, the microbiome could play a particularly important role in cases where evidence may have been transferred through skin contact but fingerprints or low amounts of human genetic material have been left behind. While the bacterium used here is primarily a component of the human gut microbiome, rather than the skin microbiome, it was used as a representative gram‐negative bacillus, and its collection may still be relevant in certain forensic case scenarios that involve evidence in the form of faeces and urine.

When the *P*. *mirabilis* was spotted directly onto each swab type, the highest average mass of microbial DNA post‐extraction was observed with the flocked swabs, followed by the positive manipulation control, dental applicators, dissolvable swabs, and finally cotton swabs. Generally, flocked and cotton swabs, which have been compared to each other numerous times in the forensic field for the recovery of human DNA, performed roughly as expected (Adamowicz et al., 2014; Bruijns et al., 2018; Viviano et al., 2018). Yet, the overall results seem counterintuitive in many respects. The flocked swabs unexpectedly yielded greater total recoveries than the positive manipulation control (comparatively 186% of the positive manipulation control recovery—Table 1), which contained no swab material. This is interesting when one notes that similar recoveries to the positive manipulation control were observed with the other low surface area groups, that is, dissolvable swabs and dental applicators (64% and 80% of the positive manipulation control recovery, respectively—Table 1). If the only factor in elution efficiency was the entrapment of sample within the swab, one would expect the no/low‐swab‐volume samples to yield comparatively greater amounts of DNA. The flocked swabs are made with nylon fibres that are positioned to keep the sample near the surface and readily available for elution. However, if the molecular composition and orientation of the swab fibres were the only factors in elution efficiency, again, one would expect similar yields from the same amount of sample deposition onto both the flocked swabs and dental applicators, as both are manufactured using flocked nylon fibres. Perhaps entrapment and swab composition are only half the story when it comes to microorganisms. We postulate that another critical factor of the swab may be a minimum surface area that allows for efficient drying or dehydration of bacterial cells, which may contribute to or assist in osmotic cell lysis, particularly because bacterial cells are protected by a peptidoglycan cell wall as well as two membranes in gram‐negative bacteria. Additionally, cells may be more dispersed over the swab surface, which may have disrupted cell adhesion and allowed better access to extraction reagents.

It should be noted that several limitations were surrounding the use of the dissolvable swabs that may have contributed to the variability and the lower‐than‐expected yields from this group. The dissolvable swabs used here were a prototype, which required manual shaving to ~ ¼ the original size before use, and extraction volumes were doubled for the dissolvable swabs so as not to saturate the system (recommendations provided by Luna Innovations Incorporated™, personal correspondence 30 November 2020). These precautions may not have been sufficient to prevent the dissolved, or partially dissolved, swab material from interfering with the bead‐based extraction process. Furthermore, the MagMAX™ DNA Multi‐Sample Ultra 2.0 Kit, which was selected for DNA extraction because it may be used to isolate DNA from a variety of cell types, including bacteria, and has the requisite guanidinium salt component for dissolvable swabs, is not routinely utilized in forensic analysis. Theoretically, the forensic use of the MagMAX™ DNA Multi‐Sample Ultra 2.0 Kit should not pose problems, assuming that it is properly and thoroughly validated.

It is recognized that another limitation was the decision to compare only two of the four swab types in the surface study. Flocked swabs were chosen for their comparatively high yields in the initial elution study. Even though higher yields in total mass were recovered from the dental applicators (2.9 × the amount of cotton) and dissolvable swabs (2.3 × the amount of cotton), yields between the three remaining swab types (dental, dissolvable, and cotton) were not statistically different from each other. Considering swab volume, the dental applicators and dissolvable swabs displayed a better release efficiency than the cotton swabs; however, due to their smaller sizes, there was also a greater risk of saturation when being used to recover materials from surfaces. Furthermore, the dental applicators were of the same general construction (flocked nylon fibres) as the flocked swabs. While cotton swabs were the least effective at releasing the *P*. *mirabilis* DNA, in terms of absolute quantity, it was suspected that they would be the most efficient at collecting biological material off of surfaces. For these reasons, and their greater use and availability in the forensic community compared to dental applicators and dissolvable swabs, cotton swabs were chosen for further evaluation in the surface study.

The flocked swabs performed comparatively well across glass, tile, and wood surfaces, but the amount of microbial DNA recovered from plastic was significantly less (Table 2). This may be due to the polypropylene composition of the plastic surface. If, as hypothesized above, an increased swab surface area contributes to bacterial cell dehydration and lysis, thus aiding in DNA recovery, this might also be occurring when samples are deposited and allowed to dry on various surfaces. The adsorption to and denaturation of DNA by plastics, particularly polypropylene, is a well‐documented phenomenon in the forensic DNA community that results in apparent DNA loss (Belotserkovskii & Johnston, 1996, 1997; Gaillard & Strauss, 1998, 2000; Kline et al., 2005; Lecerf & Le Goff, 2010; Wang et al., 2019). Cotton swabs continued to perform poorly when surface swabbing was incorporated, demonstrating no or decreased recoveries. The decrease in recoveries seen with cotton swabs was consistent with what was observed for the flocked swabs, which showed as much as a 95% decrease in yield when comparing recoveries from swabs without surface sampling to recoveries obtained following surface sampling (Table 2).

All prepared swabs, non‐porous surfaces, and surface collection swabs were allowed to air‐dry for ~2 h following their respective preparations. As all swabs and surfaces throughout the study were exposed to the same environmental conditions, any contamination or effect from other aerosolized microbes should have been equally distributed across samples. While contamination with aerosolized microbes is a possibility, the negative results observed when using cotton swabs to collect bacterial samples from the various non‐porous surfaces demonstrate that the potential effect from environmental contamination is negligible. This is a critical finding for the potential future forensic application of the microbiome as swabbed surfaces will never be completely protected from aerosol contamination between sample deposition and collection. From the forensic standpoint, systemic contamination in the form of contaminated reagents and sample‐to‐sample contamination pose bigger threats, which are typically controlled for with the processing of reagent blanks.

## 5 CONCLUSIONS

While the human DNA extraction efficiency of various swab types has been a topic of much research (Adamowicz et al., 2014; Bruijns et al., 2018; Viviano et al., 2018; Voorhees et al., 2006), sample elution is only half the story. Here, we also examine the efficiency of flocked and cotton swabs for sample recovery from various non‐porous surfaces. Additionally, except for work by Ogai et al. (2018) that compared cotton swabbing to tape lifting for collection of the microbiome, research on the optimal collection substrate for the microbiome in a forensic or non‐medical setting is scarce. While future research could focus specifically on the transfer and collection of samples relating to the skin microbiome, these data support moving toward the use of flocked swabs, and away from cotton swabs, for the collection and analysis of bacterial samples relating to forensic use of the microbiome.

## 6 ETHICS STATEMENT

None required.

## CONFLICTS OF INTEREST

None declared.

## AUTHOR CONTRIBUTIONS

**Natalie Wise:** Formal analysis (supporting); Funding acquisition (equal); Investigation (lead); Visualization (lead); Writing‐original draft (lead). **Sarah Wagner:** Conceptualization (lead); Methodology (equal). **Travis J Worst:** Methodology (equal); Resources (lead); Writing‐review & editing (supporting). **Jon Sprague:** Funding acquisition (equal); Writing‐review & editing (supporting). **Crystal Oechsle:** Formal analysis (lead); Methodology (equal); Supervision (lead); Writing‐review & editing (lead).

## Data Availability

All data generated or analysed during this study are included in this published article.
